# The Invention and Early History of the N-Localizer for Stereotactic Neurosurgery

**DOI:** 10.7759/cureus.642

**Published:** 2016-06-14

**Authors:** Russell A Brown, James A Nelson

**Affiliations:** 1 Principal Engineer, A9.com; 2 Radiology (Emeritus), University of Washington

**Keywords:** stereotactic neurosurgery, stereotactic radiosurgery, image guidance, image-guided, computed tomography, magnetic resonance imaging, positron emission tomography (pet), n-localizer, medical imaging, brain imaging

## Abstract

Nearly four decades after the invention of the N-localizer, its origin and history remain misunderstood. Some are unaware that a third-year medical student invented this technology. The following *conspectus* accurately chronicles the origin of the N-localizer, presents recently discovered evidence that documents its history, and corrects misconceptions related to its origin and early history.

## Introduction and background

**Figure 1:**
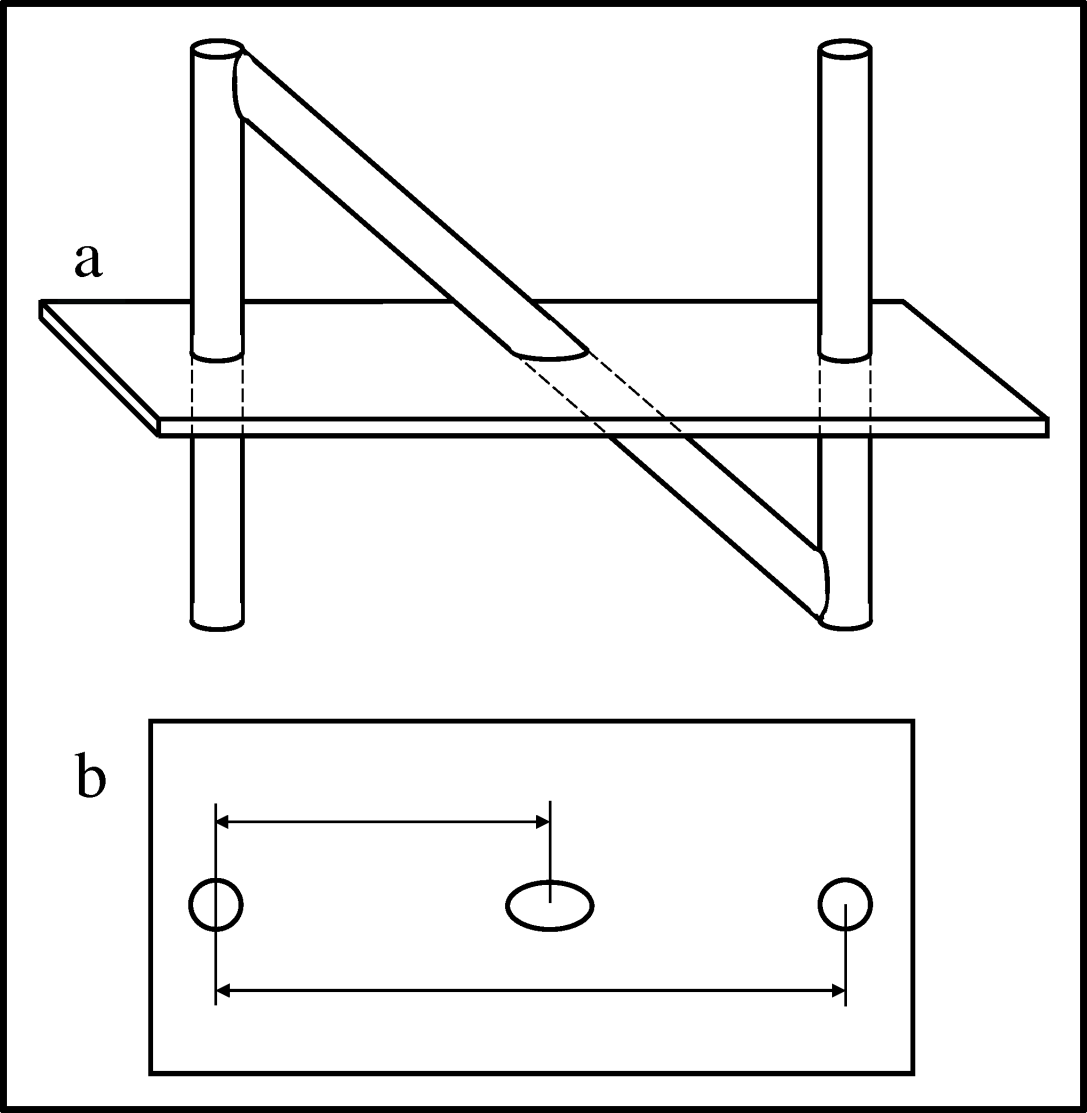
N-Localizer and Its Interaction with the Tomographic Section $\mathbf{(a)}$ Side view of the N-localizer. The tomographic section intersects the N-localizer at two vertical rods and one diagonal rod. $\mathbf{(b)}$ Tomographic image. The intersection of the tomographic section with the N-localizer produces two circles and one ellipse. The relative spacing between the centers of the ellipse and the two circles varies according to the height at which the tomographic section intersects the diagonal rod. Measuring this spacing permits calculation of the position of the tomographic section with respect to the N-localizer [2].

The N-localizer (*aka *N-bar) has become an important tool for image-guided stereotactic neurosurgery and radiosurgery. The N-localizer produces two circles and one ellipse in tomographic images obtained via computed tomography (CT), magnetic resonance (MR), or positron emission tomography (PET). The relative spacing between the ellipse and the two circles precisely determines the position of the tomographic section with respect to the N-localizer (Figure 1) [1-2].

**Figure 2:**
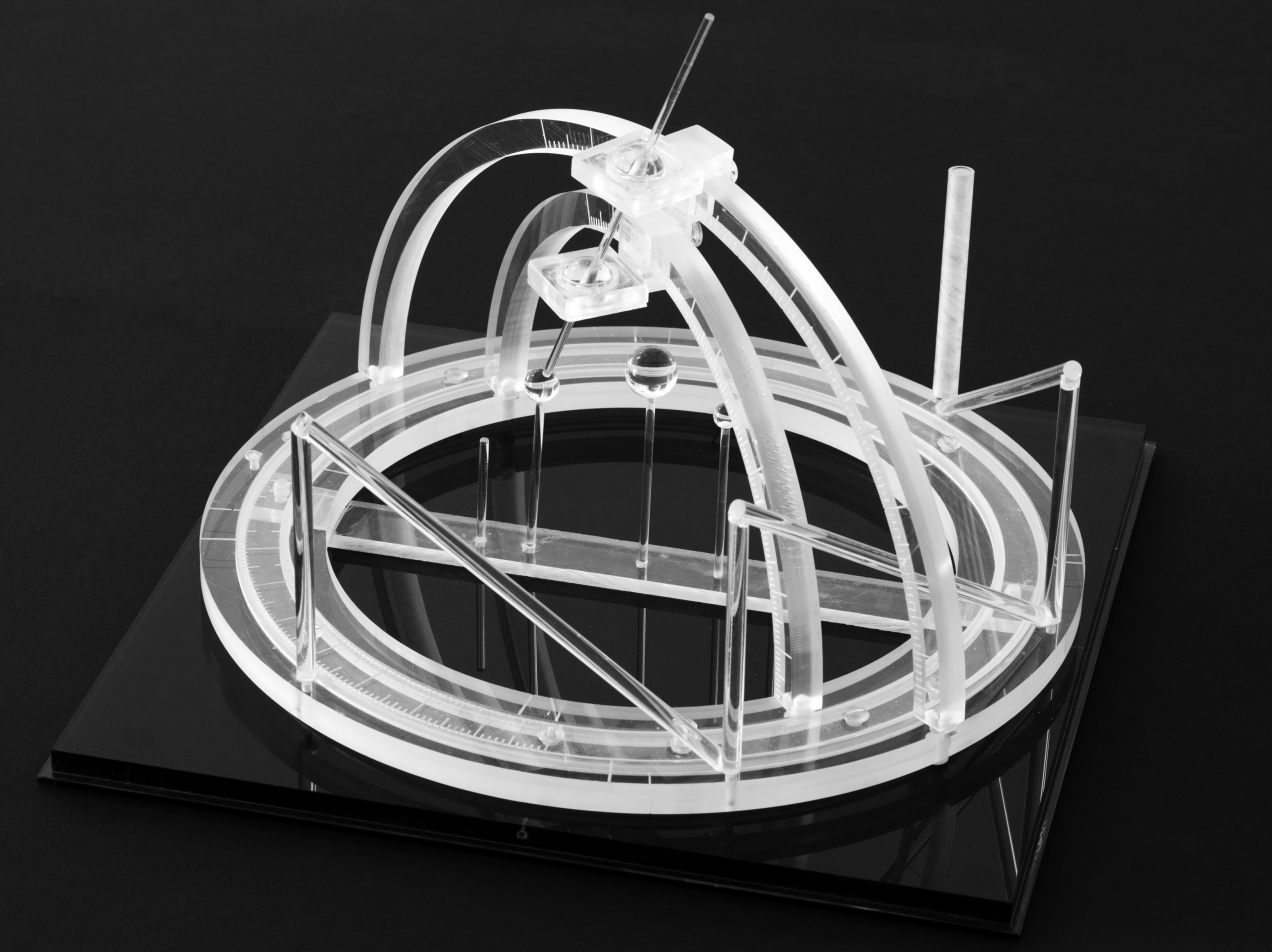
The First CT-Compatible Stereotactic Frame Brown designed and built this stereotactic frame in August 1978 in order to test the concept of the N-localizer [1]. Three N-localizers are attached to this frame and are merged end-to-end such that only seven rods are present. Because three points determine a unique plane in three-dimensional space, the locations of the centers of the three ellipses that the three N-localizers produce in a tomographic image precisely determine the position of the tomographic section with respect to the stereotactic frame [2].

Russell A. Brown invented the N-localizer in May 1978 when he was a third-year medical student and during a research elective under the supervision of James A. Nelson at the University of Utah [3]. In August 1978, Brown designed and built the first CT-compatible stereotactic frame in order to test the concept of the N-localizer (Figure 2). This stereotactic frame was presented at a joint meeting of the Western Neurological Society and the American Academy of Neurological Surgery held in Los Angeles, California in October 1978 [1] and at the INSERM Symposium on Stereotactic Irradiations held in Paris, France in July 1979 [4].

Beginning in 1979, seven different stereotactic frames incorporated the N-localizer: Brown-Roberts-Wells (BRW) [5], Pfizer [6], Todd-Wells [7], Reichert-Mundinger [8-9], Kelly-Goerss Compass [10], Leksell [11], and Cosman-Roberts-Wells (CRW) [12]. Subsequently, the N-localizer achieved widespread use in image-guided stereotactic neurosurgery and radiosurgery directed by CT [13-16], MR [17-21], and PET [22-23].

The N-localizer has assisted stereotactic radiosurgery [24-26] and neurosurgery for tumor biopsy [27-28] and resection [29], for hematoma evacuation [30-31], for cyst and abscess aspiration [31-34], for brachytherapy [31-37], for electrode placement to manage pain [31] and epilepsy [31, 38-39] and to treat Parkinson tremor [40-42], and for thalamotomy, pallidotomy, and dentatomy [31,42]. The simplicity and accuracy of the N-localizer render it an important tool for modern neurosurgery [3].

The impact of the N-localizer on stereotactic surgery and radiosurgery may be estimated from data compiled by Linskey, who counted the types of journal articles published during the years 1966 through 2003 [43]. Those data are summarized as follows [3].

Publications devoted to tumor stereotaxis were a minor fraction of all stereotactic neurosurgery publications until 1979, when the N-localizer was first described [1-2]. Beginning in 1980, the number of publications devoted to stereotactic surgery, brachytherapy or radiosurgery increased rapidly. Publications devoted to frame-based stereotactic surgery dominated until 1994, when they were overtaken by publications devoted to stereotactic radiosurgery. Publications devoted to stereotactic radiosurgery progressively increased and subsequently outnumbered publications devoted to stereotactic surgery or brachytherapy. During the three years from 2001 through 2003, publications devoted to stereotactic radiosurgery comprised roughly two-thirds of all publications devoted to cranial tumor stereotaxis.

The volume of stereotactic radiosurgery assisted by the N-localizer has continued to increase until the present day. Leksell and Lunsford affirm [44], "Statistics reported by about 80% of all Gamma Knife centers indicate that as of 2014, more than a million patients had undergone Gamma Knife surgery, and approximately 60,000 new patients undergo such surgery every year."

## Review

During the 38 years since the invention of the N-localizer, misconceptions have arisen concerning its origin and early history in relation to subsequent developments in image-guided stereotactic surgery. Those misconceptions have been discussed previously [45]. The current article presents recently discovered evidence (see Figure 9 in Appendix 6) that corroborates that previous discussion.

The first misconception maintains that the Pfizer frame was the first CT-compatible stereotactic frame and that it was constructed in 1978. Kondziolka and Lunsford of the University of Pittsburgh assert this misconception, together with their failure to discuss the prior literature, in their claim [46], "At our center, the first CT compatible stereotactic head frame, in collaboration with industry, was constructed in 1978 and utilized in 13 patients [6, 47]. *[...]* During this interval, the newly redesigned Leksell CT compatible stereotactic head frame [13] was used for dedicated brain biopsies under the direction of its inventor, Lars Leksell. Several groups were working on devices to allow accurate CT based stereotactic surgery [48].”

The above assertion presents an erroneous chronology. The Pfizer frame was neither the first CT-compatible stereotactic frame (see Figure 2) nor was it constructed and used in 1978. Instead, it was constructed and used in 1979, as per Lunsford et al., who recount [49], "In 1979, our first efforts in image-guided stereotactic surgery attempted to adapt an early-generation Leksell frame. The metallic artifacts precluded adequate computerized tomography (CT) imaging, and we subsequently developed a CT-compatible stereotactic device (Pfizer frame *[...] *) [50, 6] which was used in an initial series of 15 patients beginning in 1979." This statement is corroborated by Lunsford, Niranjan, Kassam, Khan, Amin, and Kondziolka, who state [51], "During the interval of 1979 to 1980, 13 stereotactic procedures were performed in a diagnostic scanner at our hospital." These two statements confirm that the Pfizer frame was constructed and used in 1979, not in 1978.

Further evidence that the Pfizer frame was constructed in 1979 is provided by Perry, Rosenbaum, Lunsford, Swink, and Zorub, who state [6], "The *Pfizer* stereotactic frame was made after attempts to modify the Leksell frame *[...]* proved difficult" (italics added). This evidence is corroborated by a letter from Perry to Lunsford, Rosenbaum, and Zorub [52] and a letter from Pfizer Medical Systems to its patent attorney [53]. Those letters verify that as of January 15, 1979, Perry, Rosenbaum, Lunsford, and Zorub had not yet attempted any surgery using the modified early-generation Leksell frame. Hence, the Pfizer frame, which was constructed after efforts to adapt the early-generation Leksell frame had failed, was constructed in 1979.

In addition to presenting an erroneous chronology, the above assertion [46] of Kondziolka and Lunsford disregards the fact that the CT-guidance technologies of the Leksell frame and the Pfizer frame were derivative. For both frames, the inclusion of vertical and diagonal elements originated from Brown's prior invention and description of the N-localizer. This fact is established by the articles that introduced the Leksell [13] and Pfizer [6] frames. Both articles cited one [1] of Brown's seminal articles that had introduced the N-localizer one year earlier [1-2]. Although Lunsford (with and without Kondziolka) had previously cited one or the other of Brown’s seminal articles in multiple publications [6, 31, 50-51, 54-55], these coauthors cited neither seminal article in their above assertion. Instead, they cited a later article by Roberts and Brown [48] that was published contemporaneously with the first articles from the University of Pittsburgh [6, 47] and one year after Brown’s seminal articles had introduced the N-localizer.

The second misconception maintains that investigators from Pfizer Medical Systems and the University of Pittsburgh invented the N-localizer. This misconception is asserted by Lunsford, Niranjan, Kassam, Khan, Amin, and Kondziolka, who claim [51], “During the subsequent years of training, the senior author had an opportunity to work with an innovative neuroradiologist, Arthur Rosenbaum, M.D., and an engineer, John Perry, Ph.D., who then headed the imaging division of Pfizer Medical Instruments. Together, we developed an image-guided stereotactic system using the now well-known N-localizer technology. This elegant solution was proposed by Perry et al. [6] and Rosenbaum et al. [47] independently and virtually simultaneously as publications from Brown [2] and Roberts and Brown [48] of Utah."

In the above assertion, the intended antecedent of "elegant solution" could be "image-guided stereotactic system" or "N-localizer technology." Perry et al. did propose the Pfizer image-guided stereotactic system [6], which incorporated the N-localizer, several months after Brown et al. had proposed the Brown-Roberts-Wells (BRW) image-guided stereotactic system [5]. However, the historical record shows that none of the above-mentioned individuals, with the exception of Brown, invented the N-localizer. Instead, Perry adopted the N-localizer after Brown had disclosed it to him. Documents that corroborate these facts have remained preserved in the archives of the U.S. Patent and Trademark Office for more than 30 years. The following discussion, which is based on those archives, recounts Perry's research related to image-guided stereotactic surgery and reveals the events that led to his adoption of the N-localizer.

**Figure 3:**
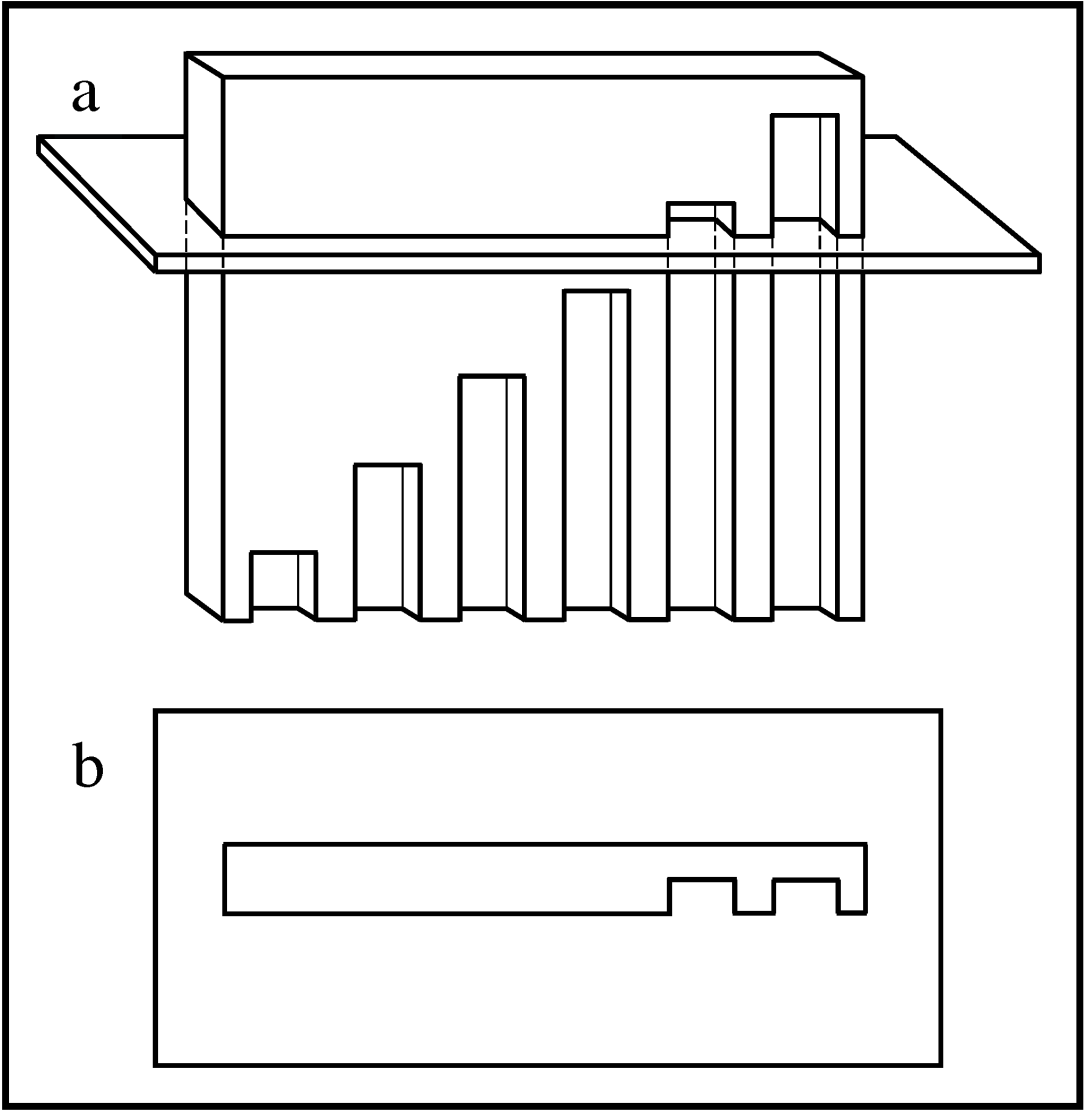
Slotted Plate and Its Interaction with the Tomographic Section $\mathbf{(a)}$ Side view of the slotted plate. The tomographic section intersects the plate into which are milled vertical slots of different lengths. The tops of the slots lie along a diagonal line. $\mathbf{(b)}$ Tomographic image. The intersection of the tomographic section with the slotted plate produces a variable number of notches. The number of notches depends on the height at which the tomographic section intersects the plate. Counting the number of notches permits estimation of the position of the tomographic section with respect to the slotted plate.

**Figure 9:**
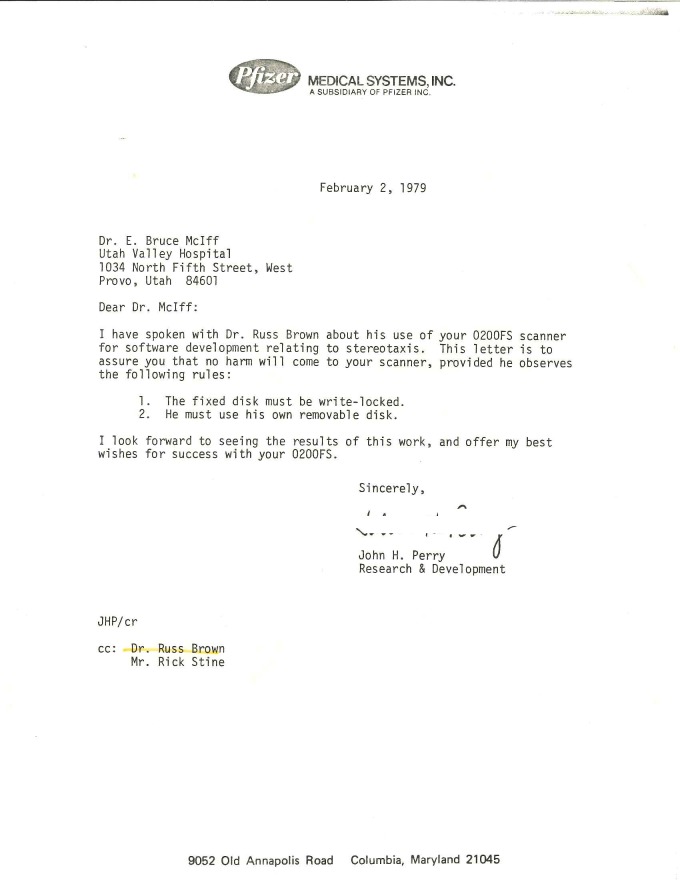
Appendix 6: John Perry Letter, p. 1, February 2, 1979

Prior to the invention of the N-localizer, Lee, Villafana, and Lapayowker had reported a method for estimating the position of a tomographic section with respect to patient anatomy [56-57]. Their method involved a plate into which were milled vertical slots whose tops lay along a diagonal line (Figure 3).

Documents from the archives of the U.S. Patent and Trademark Office indicate that as of January 15, 1979, Perry, Rosenbaum, Lunsford, and Zorub had attached three slotted plates to a Leksell frame [52-53]. In principle, three slotted plates enabled the calculation of the position of a tomographic section with respect to a stereotactic frame, similar to the manner in which three N-localizers enable this calculation (Figure 2).

In practice, however, the slotted plate was susceptible to error as a result of the discrete or quantized nature of the slots. Perry observed that it was necessary to count carefully the numerous notches that were visible in the tomographic image, because any miscount would give rise to errors in the subsequent calculation of the position of the tomographic section with respect to the stereotactic frame [52]. Moreover, the partial volume effect [58-59], which derives from the several-millimeter thickness of the tomographic section, impeded accurate counting of the notches, because any slot that passed partially into but not entirely through the tomographic section would produce an only faintly visible notch. For these reasons, the slotted plate was vulnerable to human error and hence was unsuitable for clinical use. The N-localizer avoids these quantization problems and the attendant possibility of computational errors by virtue of the continuous nature of the N-localizer's rods.

Perry's earliest description of the slotted plate, and indeed the earliest record of his involvement with image-guided stereotactic surgery, was in his letter dated January 15, 1979, and addressed to his collaborators, Lunsford, Rosenbaum, and Zorub at the University of Pittsburgh [52]. Perry's letter describes three slotted plates attached to a stereotactic frame and provides instructions for using computer software in conjunction with those slotted plates to calculate the position of a tomographic section with respect to that stereotactic frame. Well before the date of Perry's letter, Brown had already invented the N-localizer [60], built the first CT-compatible stereotactic frame [61], and presented his results to the Western Neurological Society and the American Academy of Neurological Surgery [1].

On January 25, 1979, Brown spoke by phone with one of Perry's coworkers at Pfizer Medical Systems and learned that Perry's research concerned image-guided stereotactic surgery [62]. The following day, Pfizer Medical Systems sent to its patent attorney a letter that included a photo of a Leksell frame to which three slotted plates were attached and a photo of a CT image of the Leksell frame and slotted plates [53]. A few days later, Brown spoke by phone with Perry and disclosed the N-localizer to him [63]. Perry et al. subsequently abandoned the slotted plate, adopted the N-localizer, and incorporated it into the Pfizer CT-compatible stereotactic frame [6].

Perry's earliest description of the N-localizer was in his application to the U.S. Patent and Trademark Office dated April 13, 1979 [64]. When challenged by Brown via a Patent Interference proceeding before the U.S. Patent and Trademark Office, Perry failed to provide any evidence whatsoever of having invented the N-localizer. Moreover, Brown's invention of the N-localizer [60] had preceded Perry's earliest involvement with image-guided stereotactic surgery [52] by eight months. Consequently, Perry conceded “priority of invention” to Brown [65], and the U.S. Patent and Trademark Office awarded to Brown patent protection for the N-localizer and for other significant aspects of image-guided stereotactic surgery [66]. The documents [1, 52-53, 60-62, 65] that the U.S. Patent and Trademark Office examined prior to awarding patent protection to Brown instead of Perry are a matter of public record. Those documents may be obtained from the U.S. Patent and Trademark Office by requesting a copy of the folder for Interference No. 101267. In order to facilitate access to those documents, copies are included in the Appendices to this article.

## Conclusions

Brown invented the N-localizer and built the first CT-compatible stereotactic frame in 1978. The N-localizer has become an important tool for modern neurosurgery and has achieved widespread use in image-guided stereotactic neurosurgery and radiosurgery directed by CT, MR, and PET. Beginning in 1979, seven different stereotactic frames incorporated the N-localizer. For each frame, the inclusion of the N-localizer was derivative and originated from Brown's research.

## Figures and Tables

**Figure 4:**
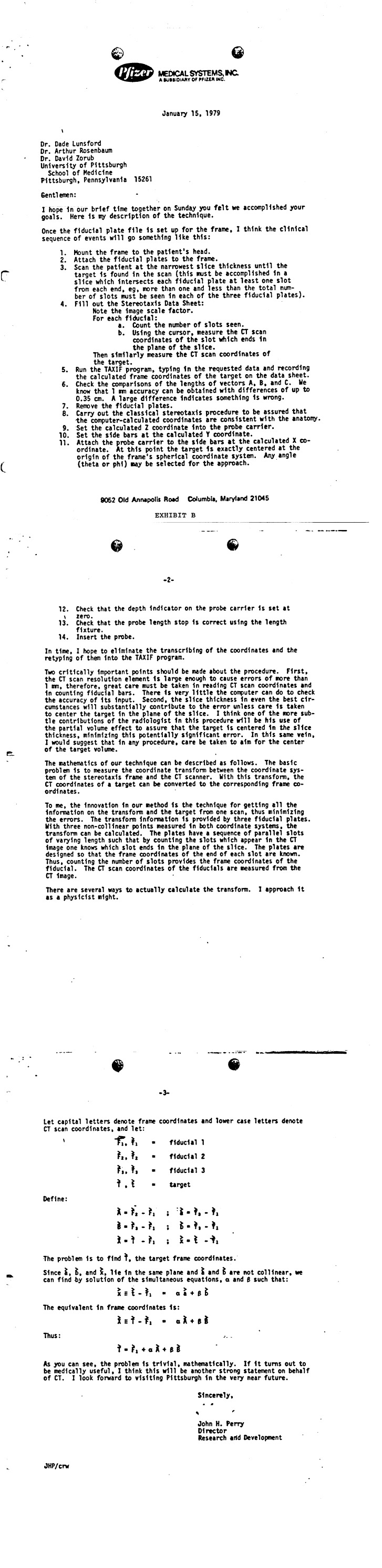
Appendix 1: John Perry Letter, pp. 1-3, January 15, 1979

**Figure 5:**
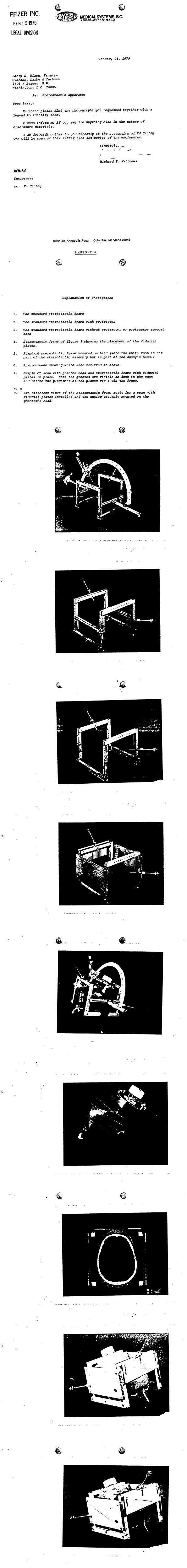
Appendix 2: Richard Matthews Letter, pp. 1-7, January 26, 1979

**Figure 6:**
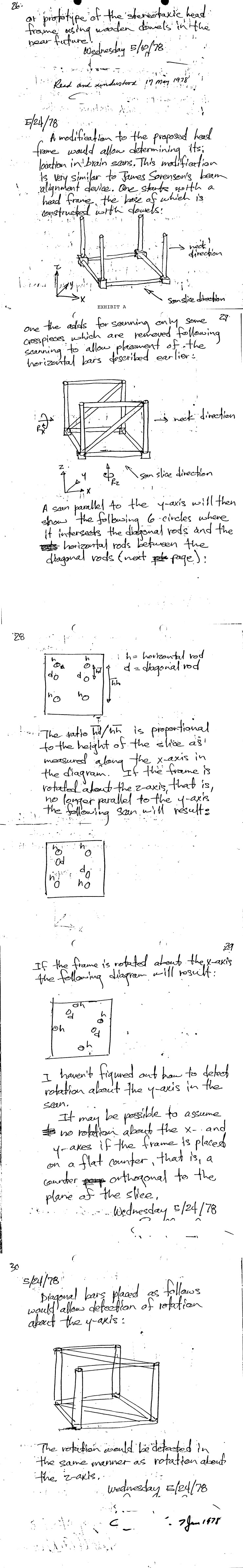
Appendix 3: Russell Brown Notebook 1, pp. 26-30, May 24, 1978

**Figure 7:**
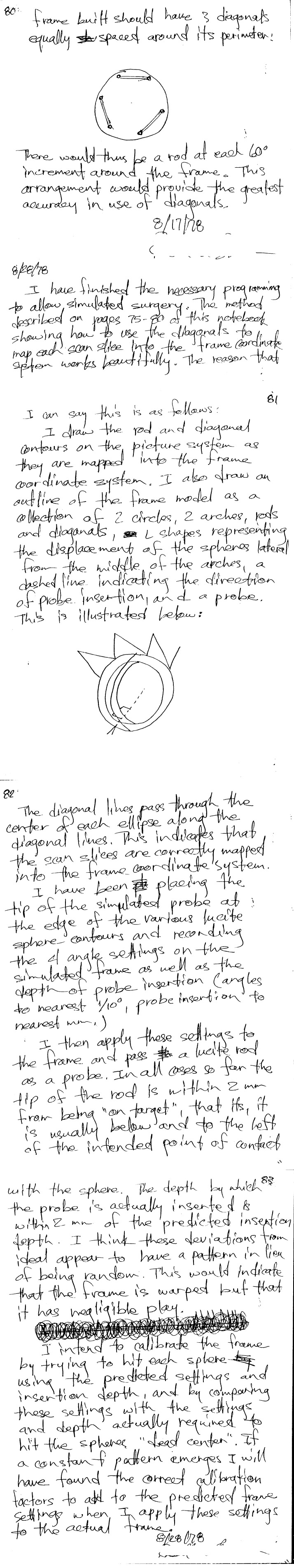
Appendix 4: Russell Brown Notebook 1, pp. 80-83, August 28, 1978

**Figure 8:**
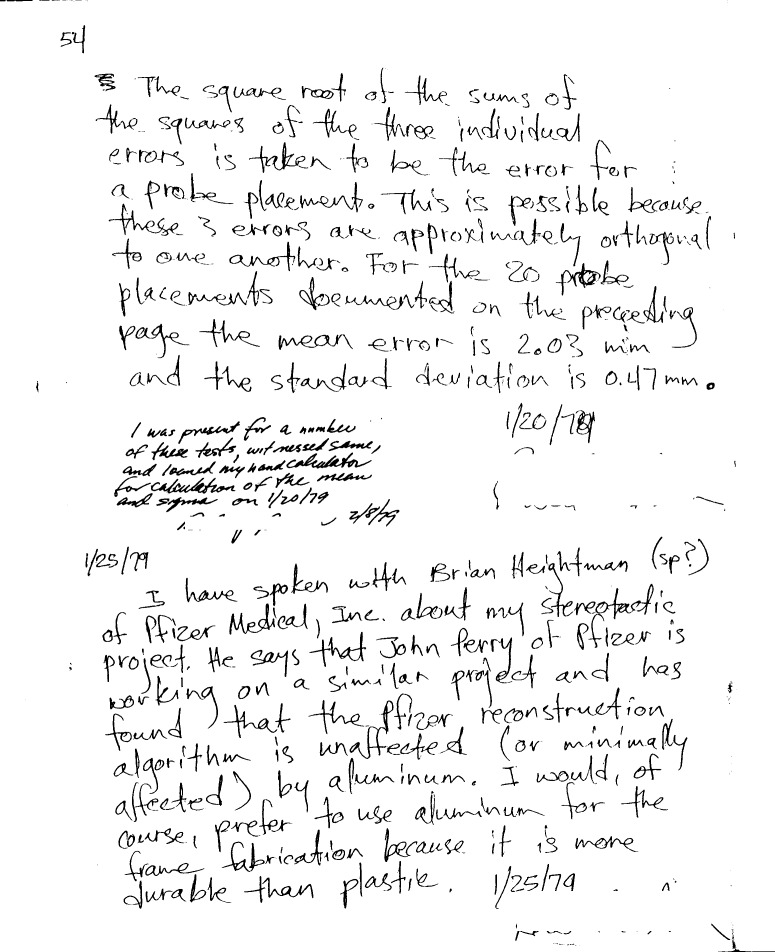
Appendix 5: Russell Brown Notebook 2, p. 54, January 25, 1979

**Figure 10:**
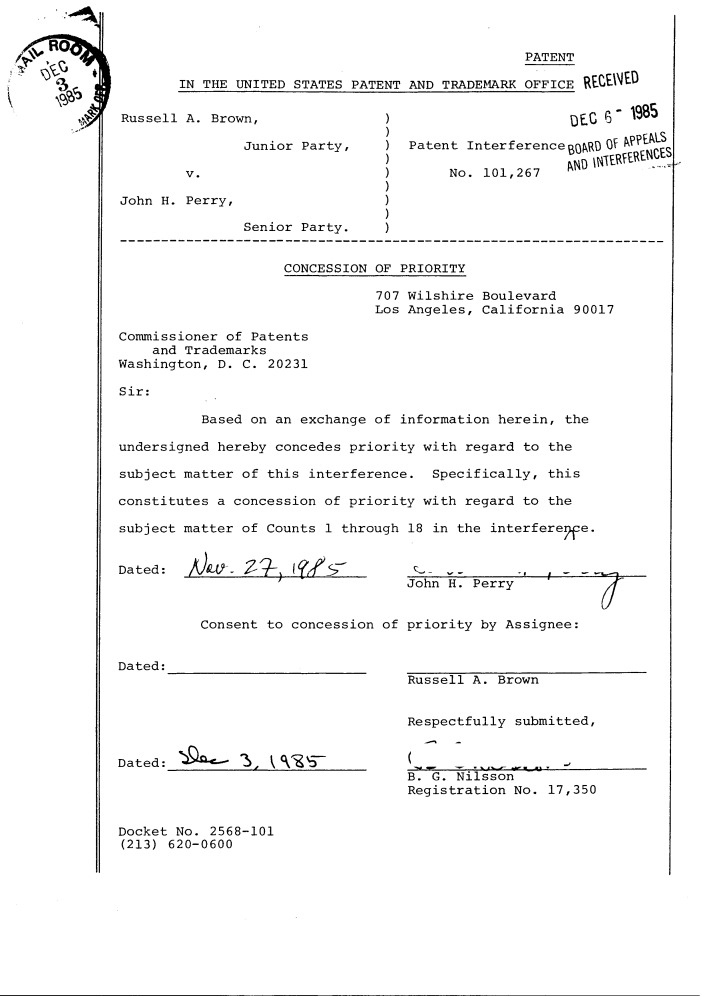
Appendix 7: John Perry Concession of Priority, November 27, 1985
